# The Incidence and Risk Factors of Cholelithiasis Development After Bariatric Surgery in Saudi Arabia: A Two-Center Retrospective Cohort Study

**DOI:** 10.3389/fsurg.2020.559064

**Published:** 2020-10-22

**Authors:** Mohammed A. Aldriweesh, Ghadeer L. Aljahdali, Edi A. Shafaay, Dalal Z. Alangari, Nawaf A. Alhamied, Hadeel A. Alradhi, Amirah S. Yaqoub, Sami El-Boghdadly, Omar S. Aldibasi, Abdallah A. Adlan

**Affiliations:** ^1^College of Medicine, King Saud bin Abdulaziz University for Health Sciences, Riyadh, Saudi Arabia; ^2^College of Applied Medical Sciences, King Saud bin Abdulaziz University for Health Sciences, Riyadh, Saudi Arabia; ^3^Department of Surgery, King Abdulaziz Medical City, Riyadh, Saudi Arabia; ^4^College of Dentistry, King Saud bin Abdulaziz University for Health Sciences, Riyadh, Saudi Arabia; ^5^Department of Biostatistics and Bioinformatics, King Abdullah International Medical Research Center, Riyadh, Saudi Arabia; ^6^Department of Bioethics, King Abdullah International Medical Research Center, Riyadh, Saudi Arabia

**Keywords:** gallstones, weight loss, cholecystecomy, BMI—body mass index, LSG, laparoscopic sleeve gastrectomy

## Abstract

**Background:** Rapid weight loss after bariatric surgery is a known risk factor for cholelithiasis development. This study aimed to estimate the incidence of cholelithiasis following bariatric surgery among morbidly obese patients who underwent bariatric surgery.

**Methods:** This is a retrospective cohort study of all morbidly obese patients who underwent bariatric surgery in King Abdulaziz Medical City (Riyadh, Saudi Arabia) or King Abdulaziz Hospital (Al Ahsa, Saudi Arabia) between January 2015 and December 2018. Patients with a history of cholecystectomy or previous bariatric surgery were excluded. We estimated the incidence rate of cholelithiasis among the cohort. We also examined the associated risk factors of cholelithiasis development.

**Results:** The study cohort contained 490 patients (38.7% males; 61.43% females) with a mean age of 36.87 ± 11.44 years. Most patients (58.54%) were followed up for 12 months. The incidence of cholelithiasis post-operation was 6.53% (*n* = 32). The average period of cholelithiasis formation was 12–24 months. The percentage of total weight loss (TWL%) was significantly associated with the development of cholelithiasis post-operatively.

**Conclusion:** A significant association was found between weight loss following bariatric surgery and the incidence of cholelithiasis. Gender, age, and comorbidities were not associated with the formation of cholelithiasis. We recommend regular follow-up appointments with thorough patient education about gradual weight loss to reduce the risk of developing cholelithiasis.

## Introduction

Obesity is one of the leading global health burdens, and its prevalence has increased between 1998 and 2013 from 28.8 to 36.9% in men and from 29.8 to 38.0% in women (1). Obesity is associated with additional complications and diseases, highlighting the ever-growing need for effective solutions to obesity. Many morbidly obese individuals undergo bariatric surgery after repeatedly failed trials of diet and physical activity. Bariatric surgery is used to promote weight loss by restricting the amount of food that can be held in the stomach.

Bariatric surgery is performed using minimally invasive techniques (2). The types of bariatric surgeries include laparoscopic sleeve gastrectomy (LSG), Roux-en-Y gastric bypass, and gastric banding. The International Federation for the Surgery of Obesity and Metabolic Disorders (IFSO) reported a total of 833,687 procedures worldwide in 2019. Among these procedures, LSG was the type most commonly performed (3). Although IFSO has reported an increased number of bariatric surgeries due to its safety and effectiveness, it can nevertheless have side effects and complications. For example, bariatric surgery was found to increase the risk of cholelithiasis.

Recent studies have measured the incidence rate of cholelithiasis following different types of bariatric surgeries. Globally, the incidence varies between ~2 and ~50% (4–8), whereas locally, the incidence rate is 2.3–3.5% (9, 10). Some reports compared the complications of bariatric surgery and found that patients who underwent LSG are less prone to develop cholelithiasis (11, 12). Furthermore, rapid weight loss after bariatric surgery is strongly correlated with cholelithiasis development (13, 14). A recently published local study in Saudi Arabia reported a cholelithiasis incidence rate of 3.5% after bariatric surgery. It also found that cholelithiasis development was associated with rapid weight loss (10). However, further studies are needed. In this study, we aimed to measure the incidence rate of cholelithiasis after bariatric surgery in two tertiary centers in Saudi Arabia.

## Materials and Methods

### Study Design and Settings

This retrospective cohort study was conducted in National Guard medical cities: King Abdulaziz Medical City—Riyadh (KAMC-RYD) and King Abdulaziz Hospital—Ahsa (KAH-A). The study covered a period from January 2015 to December 2018. The National Guard of Health Affairs consists of multiple medical complexes scattered over the main cities of Saudi Arabia. KAMC-RYD is the first and largest medical city, with a bed capacity of 1501 beds. In 2004, a university for health sciences was established, which incorporated an academic aspect to KAMC-RYD. KAH-A also serves a significant role in medical services in Al Ahsa, with a bed capacity of ~400 beds. This study was designed to identify the incidence rate of cholelithiasis after bariatric surgery and explore possible risk factors for the development of cholelithiasis post-surgery. This study included all patients who underwent bariatric surgery and followed them up to the last documented appointment.

### Study Subjects

All patients who underwent bariatric surgery in KAMC-RYD or KAH-A from January 2015 to December 2018 were included in this study. Inclusion criteria consisted of age ≥ 18 years and admitted for bariatric surgery due to morbid obesity. The exclusion criteria consisted of the presence of cholelithiasis before surgery, history of cholecystectomy, and history of previous bariatric surgery. The sampling technique used was purposive sampling.

### Data Collection

The electronic medical records of patients who met the inclusion criteria were reviewed. These patients were followed from the day of surgery until the last documented follow-up. Follow-up intervals were 0–1, 1–2, and >2 years. We collected data on the age, educational level, smoking status, laboratory results, including low-density lipoprotein (LDL), high-density lipoprotein (HDL), total triglycerides (Trig), total bilirubin (TBili), weight, height, body mass index (BMI), total weight loss percentage (TWL%), and excess BMI loss percentage (EBMI%), and comorbidities such as diabetes mellitus (DM), hypertension (HTN), dyslipidemia (DLP), and obstructive sleep apnea (OSA).

### Statistical Analysis

Data were analyzed using SAS v. 9.4 (SAS Institute Inc., Cary, NC). Descriptive analysis of the study variables was applied as percentages and frequencies for categorical variables (e.g., gender), while the numerical variables (e.g., age) were analyzed as mean and standard deviation. Inferential statistics were applied to compare risk factors between gallstone and non-gallstone patients based on the independent *t*-test and chi-square tests for continuous and categorical variables, respectively. ANOVA was performed to compare the TWL% and EBMIL% among the gallstone and non-gallstone groups at the time of the follow-up. A *p*-value of < 0.05 was considered significant.

### Ethical Approval

The study received approval from the Institutional Review Board (IRB) committee (RC18/273/R) of King Abdullah International Medical Research Center (KAIMRC) at the Ministry of National Guard Health Affairs.

## Results

From January 2015 to December 2018, a total of 888 patients underwent bariatric surgery, 490 of which met the inclusion criteria. The mean age was 36.87 ± 11.44 years. Males accounted for 38.7% (*n* = 189) while females account for 61.43% (*n* = 301) of the sample. The rate of comorbidities was as follows: DM in 29.59% (*n* = 145), HTN in 25.51% (*n* = 125), and DLP in 22.24% (*n* = 109) of the patients. The majority of the patients underwent LSG (*n* = 434; 88.57%), and the follow-up intervals were 0–1 year (*n* = 233; 58.54%), 1–2 years (*n* = 91; 22.86%), and >2 years (*n* = 74; 18.59%). Baseline characteristics are shown in Table 1.

**Table 1:** Baseline characteristics of bariatric surgery patients.

**Variable**	***N* (%)/Mean ± SD**
**Age**	36.87 ± 11.44
**Gender** Male Female	189 (38.7%) 301 (61.43)
**Smoking** Non-smoker History of smoking or smoker	408 (87.74%) 57 (12.26%)
**Comorbidity** Diabetes mellitus Hypertension Dyslipidemia Obstructive sleep apnea	145 (29.59%) 125 (25.51%) 109 (22.24%) 65 (13.27%)
**Type of surgery** Laparoscopic sleeve gastrectomy RYGB and others^a^	434 (88.57%) 56 (11.43)
**Duration of follow-up** 0–1 Year 1–2 Years >2 Years	233 (58.54%) 91 (22.86%) 74 (18.59%)
**Pre-surgery laboratory^b^** HDL (mmol/L) LDL (mmol/L) Trig (mmol/L) TBili (μmol/L)	1.13 ± 0.25 3.16 ± 0.91 1.47 ± 0.72 9.11 ± 4.75
**Pre-surgery measurement** Pre-weight (kg) Pre-height (m) Pre-BMI (kg/m^2^)	123.31 ± 21.89 1.63 ± 0.09 46.15 ± 6.94
**Post-surgery measurement** Post-weight (kg) Post-Height (m) Post-BMI (kg/m^2^) TWL (%)^c^ EBMIL (%)^d^	88.62 ± 21.75 1.63 ± 0.09 33.20 ± 7.41 0.28 ± 0.12 0.64 ± 0.31
**Cholelithiasis** Yes No	32 (6.53%) 458 (93.47%)
**Type of cholelithiasis** Symptomatic Asymptomatic	23 (89.82%) 17 (4.33%)

a *RYGB and Others, Roux-en-Y gastric band, gastric bypass, adjustable gastric band*.

b *HDL, high-density lipoprotein (N > 1.5 mmol/L); LDL, low-density lipoprotein (N < 1.8 mmol/L); Trig, triglycerides (N < 1.7 mmol/L); TBili, total bilirubin serum (N < 20.1 μmol/L)*.

c *TWL%, total weight loss percentage*.

d *EBMIL%, excess body mass index loss percentage*.

Laboratory test results and body measurements such as pre- and post-operative weight, height, and BMI are shown in Table 1. Of the 490 patients, 6.53% (*n* = 32) developed cholelithiasis post-operatively. The majority (89.82%) of patients were asymptomatic (Table 1). The mean age was 34.67 ± 9.84 years. Of these 490 patients, 6.98% (*n* = 21) who developed cholelithiasis were females, and 5.82% (*n* = 11) were males (Table 2).

**Table 2:** Association between cholelithiasis and possible risk factors.

**Variable**	**Cholelithiasis** ***N*** **(%)/Mean** **±** **SD**		***P***
	**Yes**	**No**	
**Age**	34.67 ± 9.84	37.03 ± 11.53	0.26
**Gender**			
Male	11 (5.82%)	178 (94.18%)	0.614
Female	21 (6.98%)	280 (93.02%)	
**Smoking**			
Non-smoker	26 (6.37%)	382 (93.63%)	0.85
History of smoking or smoker	4 (7.02%)	53 (92.98%)	
**Diabetes mellitus**			
Yes	10 (6.90%)	135 (93.10%)	0.83
No	22 (6.38%)	323 (93.62%)	
**Hypertension**			
Yes	8 (6.40%)	341 (93.425)	0.94
No	24 (6.58%)	117 (93.60%)	
**Dyslipidemia**			
Yes	6 (5.50%)	103 (94.50%)	0.62
No	26 (6.82%)	355 (93.18%)	
**Obstructive sleep apnea**			
Yes	3 (4.62%)	62 (95.38%)	0.50
No	29 (6.82%)	396 (93.18%)	
**Type of surgery**			
LSG	26 (5.99%)	408 (94.01%)	0.178
RYGB and others	6 (10.71%)	50 (89.29%)	
**Pre-surgery laboratory**			
HDL (mmol/L)	1.11 ± 0.18	1.13 ± 0.25	0.74
LDL (mmol/L)	3.02 ± 0.68	3.17 ± 0.92	0.44
Trig (mmol/L)	1.3 ± 0.64	1.48 ± 0.72	0.50
TBili (μmol/L)	9.4 ± 4.9	9.09 ± 4.74	0.71
**Pre-surgery measurement**			
Pre-weight (kg)	122 ± 24.14	123 ± 21	0.92
Pre-height (m)	1.6 ± 0.08	1.6 ± 0.09	0.44
Pre-BMI (kg/m^2^)	47.52 ± 7.13	46.06 ± 6.9	0.24
**Post-surgery measurement**			
Post-weight (kg)	80.73 ± 16.69	89.18 ± 21.9	0.03*
Post-height (m)	1.6 ± 0.09	1.6 ± 0.09	0.05*
Post-BMI (kg/m^2^)	31.4 ± 5.46	33.33 ± 7.52	0.06*
TWL (%)	0.33 ± 0.11	0.27 ± 0.12	0.007*
EBMIL%	0.72 ± 0.23	0.63 ± 0.31	0.05*

*The bold values showed statistical significance (A p < 0.05 was considered significant)*.

*P-value shows the statistical significance as obtained from the independent t-test and chi-square tests for continuous and categorical variables, respectively*.

**Figure 1:**
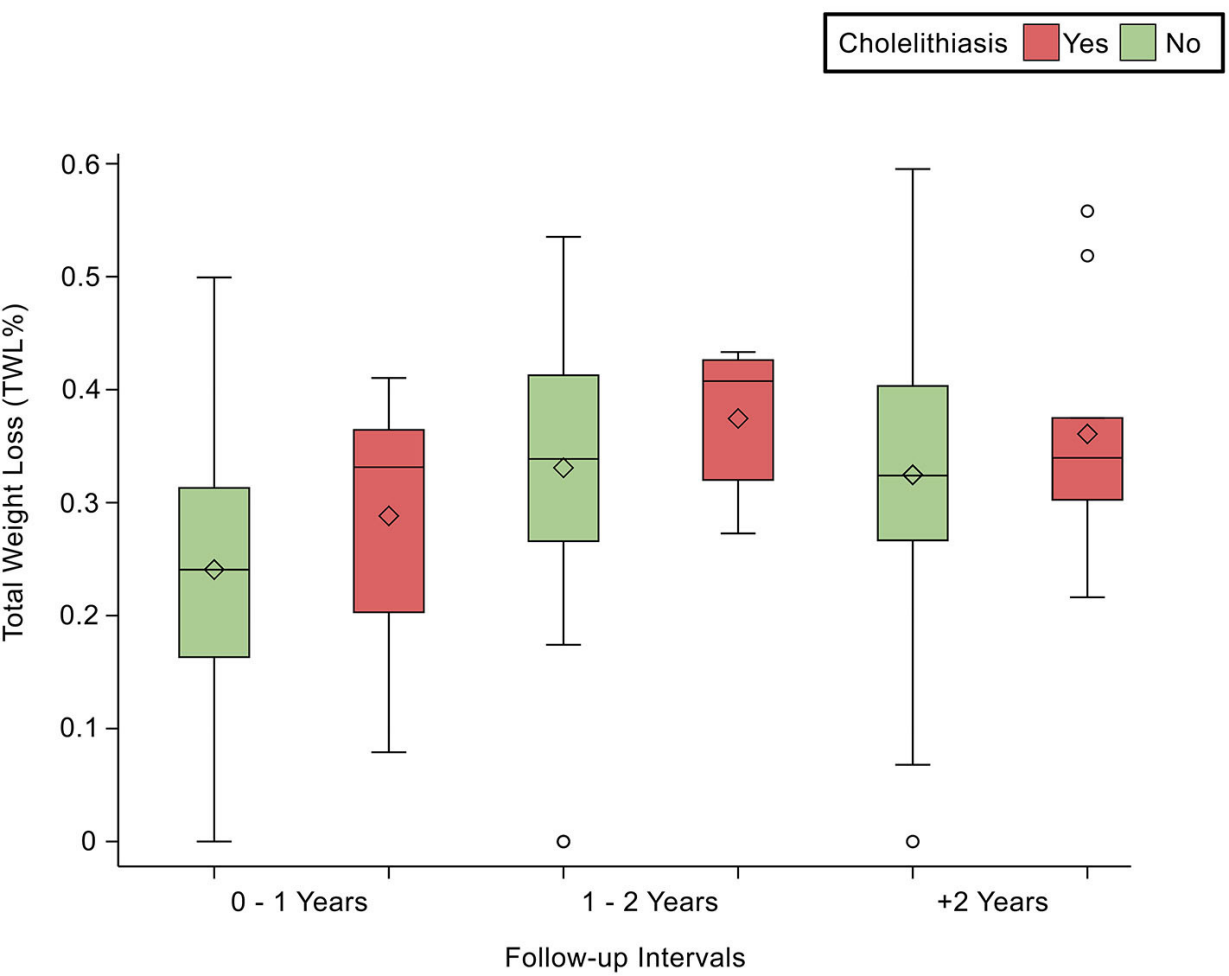
Demonstrates the statistically significant relationship between TWL (%) and follow-up intervals, *p* < 0.0001.

**Figure 2:**
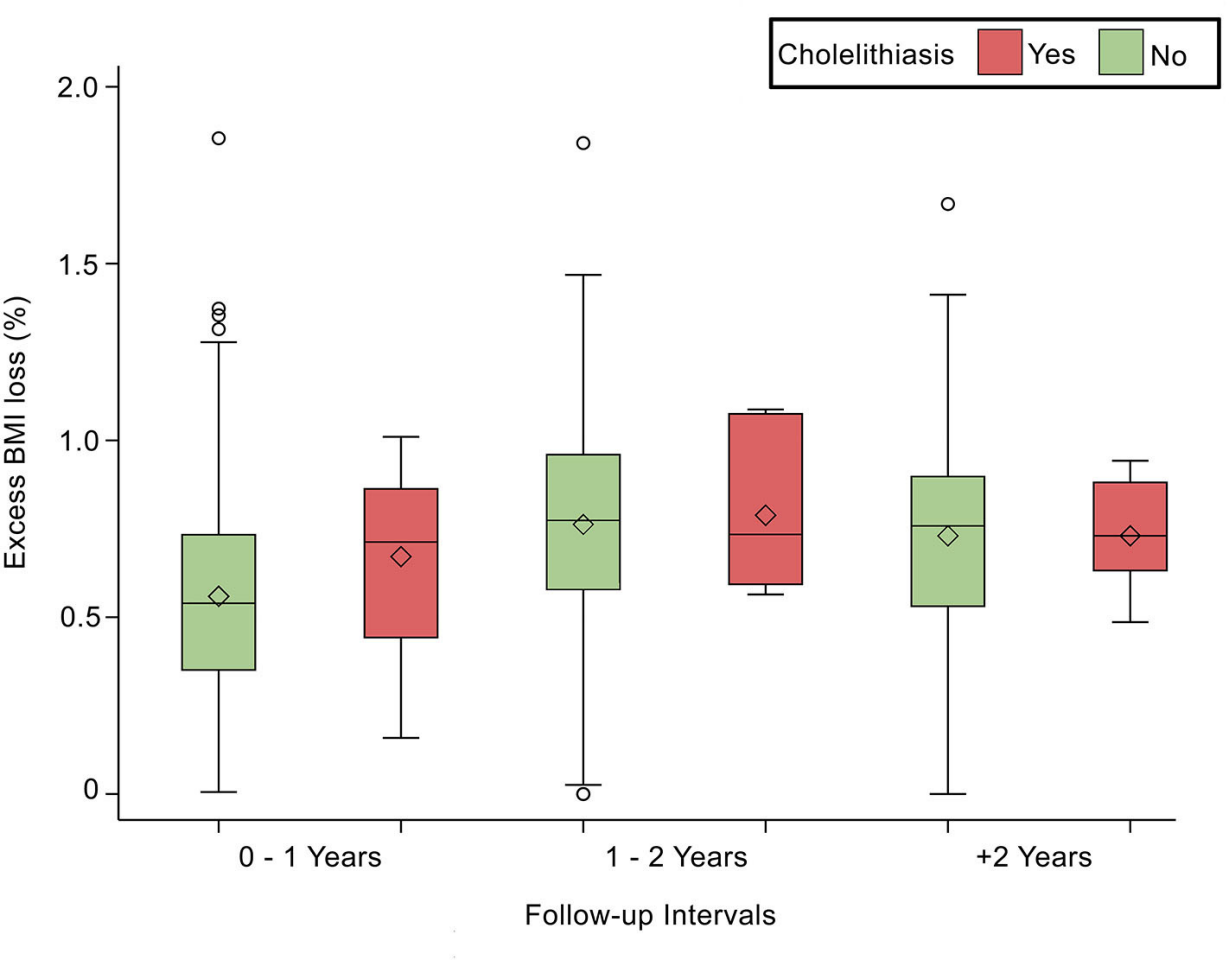
Depicts the statistically significant relationship between Excess BMI loss (%) and follow-up intervals, *p* < 0.0001.

Comorbidities were not significantly associated with cholelithiasis; DM was found in 6.90% (*n* = 10; *p* = 0.83), HTN in 6.40% (*n* = 8; *p* = 0.94), and DLP in 5.50% (*n* = 6; *p* = 0.62) of the sample. At the same time, laboratory tests were not significantly associated with cholelithiasis; HDL was found as (1.11 ± 0.18 mmol/L; *p* = 0.74), LDL (3.02 ± 0.68 mmol/L; *p* = 0.44), Trig (1.3 ± 0.64 mmol/L; *p* = 0.50), and TBili as (9.4 ± 4.9 μmol/L; *p* = 0.71). Post-operative body measurements: the post-weight average was (80.73 ± 16.69 kg; *p* = 0.03), and the post-BMI average was (31.4 ± 5.46 kg/m2; *p* = 0.06). The duration of cholelithiasis formation was significant at the >2-year follow-up (*p* = 0.003). Higher TWL% was significantly associated with cholelithiasis development post-operatively (*p* = 0.007), whereas EBMI% was not statistically significant (Table 2). Nevertheless, TWL% and EBMIL% were both significantly correlated with cholelithiasis incidence across different follow-up periods (Figures 1, 2).

## Discussion

Our study found a post-bariatric surgery cholelithiasis incidence of 6.53%, which is similar to the results of other studies, although some studies report rates up to 50% (5, 8, 10). Two studies calculated local incidence rates of 2.3 and 3.5%, respectively (9, 10). Our study revealed a higher proportion of LSG procedures relative to other bariatric procedures. Globally, incidence rates differ due to many factors. Gender, for instance, is reported as one of the risk factors for cholelithiasis development (8, 15). However, our study found no significant association between cholelithiasis and gender.

Similarly, other reports found that gender was not associated with cholelithiasis after bariatric surgeries (6, 10, 16). However, others have shown that gender is a statistically significant risk factor for cholelithiasis development (8, 17). Besides gender, age is a known risk factor; one study observed a higher mean age in patients who developed cholelithiasis (18); however, data on age and cholelithiasis development remain scarce.

Another risk factor is BMI, which plays a crucial role in the underlying pathophysiology of cholelithiasis development. A BMI of ≥40 kg/m^2^ is significantly associated with post-operative cholelithiasis (16). It has been hypothesized that a chronically elevated BMI is associated with cholesterol predominance, which, in turn, leads to cholelithiasis (19). Nevertheless, our study did not find an association between BMI and cholelithiasis.

The type of bariatric surgery has also been linked to the incidence of cholelithiasis. This is due to the complexity of the procedure and whether the procedure is restrictive or malabsorptive. Another study found a 6.2% incidence rate of cholelithiasis after bariatric surgery, and that the type of bariatric procedure affects incidence rates: Roux-en-Y gastric bypass was found to have an incidence rate of 14.5%, which is markedly higher than that of LSG, laparoscopic gastric band, or mini-gastric bypass (4.4, 4.1, and 7.5%, respectively) (20). The latter carries a higher risk due to anatomical changes that intervene with the bile signaling, movement, and saturation (18). Unfortunately, our study cohort had a much higher proportion of LSG than other bariatric procedures; therefore, we were unable to predict statistical associations between type of bariatric surgery and cholelithiasis. Our study found no association between cholelithiasis and comorbidities, which is similar to the findings of other recent studies (9, 13, 21). In another study, HTN was found to be a protective factor, unlike other comorbidities (21). This lack of consensus leaves a large gap in the medical literature with respect to comorbidities and risk factors.

Of note, a concern should be raised about metabolic syndrome's effect on post-operative outcomes since bariatric surgery becomes the definitive treatment (22–24). Namely, that metabolic syndrome is closely associated with obesity, DM, and HTN. Upon observation, we found many patients with metabolic syndrome in our cohort, yet further analysis is needed. A study found that patients with metabolic syndrome had more deficiencies other than did patients without metabolic syndrome (25). However, hyperinsulinemia appears to be an independent risk factor for cholelithiasis development after bariatric surgery (15, 26).

Rapid weight loss is considered one of the most critical risk factors for cholelithiasis development after bariatric surgery. As found in other studies, rapid weight loss was statistically associated with a higher incidence of cholelithiasis (9, 13, 21). Likewise, our study revealed an association between rapid weight loss and cholelithiasis formation. Generally, obesity and rapid weight loss are modifiable risk factors for the development of cholelithiasis. Rapid weight loss—defined as weight loss exceeding 1.5 kg per week—is perilous and increases the risk for cholelithiasis development, especially during the first post-operative year (15, 27, 28).

Contrary to expectations, our study found no significant association between incidence rates and the first post-operative year. As per our results, higher TWL% or EBMIL% is significantly correlated to the development of cholelithiasis (33 and 72%, respectively). Additionally, we found that follow-ups occurring within 1–2 years post-operatively have the highest TWL% and EBMI%. One study found that 28.94% loss of weight within the first 6 months post-operation was significantly associated with cholelithiasis (10). Our results are consistent with other international reports that found that cholelithiasis is strongly associated with weight loss greater than 25 or 30%, respectively (13, 14). Surprisingly, some studies reported no association between the proportion of weight lost and cholelithiasis (6, 21, 29).

Current guidelines consist of perioperative radiological and laboratory tests to assess possible risks (30–32). If cholelithiasis were discovered beforehand, concomitant cholecystectomy would be performed as prophylaxis (30–32). However, a lack of consensus remains on this topic. A recent study found that the risk of developing cholelithiasis after LSG in asymptomatic patients is close to the healthy population, recommending observation only in such cases (33). Another method for preventing cholelithiasis is by prescribing ursodeoxycholic acid as prophylaxis for the first 6 months post-operatively, yet there is insufficient evidence to support its efficacy. As prevention of cholelithiasis development, studies suggested avoiding the use of medications that might increase the risk of biliary dysfunction such as gemfibrozil or octreotide (34, 35).

Additionally, a perioperative patient education program, including a multidisciplinary and patient-centered approach, can significantly increase the long-term compliance of patients (32). According to the guidelines, the multidisciplinary team must include a registered dietitian who is aware of the diet required by different types of bariatric surgeries in order to help guide the patient's post-operative diet, since gallstone frequency appears to be related to weight control (32). Furthermore, a healthy-eating education should be initiated early after bariatric surgery (32, 36).

## Conclusion

Our study found a strong correlation between the amount of weight loss following bariatric surgery and the incidence of cholelithiasis. In other words, high BMI preoperatively and rapid weight loss over a short period increase the risk of developing cholelithiasis post-operatively. Gender, age, and comorbidities were not associated with the formation of cholelithiasis. Although the mechanism of cholelithiasis formation post-bariatric surgery is not clearly understood, we recommend the following: (1) to reevaluate the practice of concomitant cholecystectomy in asymptomatic patients undergoing bariatric surgeries, (2) to increase the quality of care and place emphasis on patient education by complying to the multidisciplinary team instructions, and (3) to optimize the management of bariatric surgery to minimize the risk of developing cholelithiasis by suggesting the use of prophylactic cholagogue and choleretic agents preoperatively. This research reflects that cholelithiasis incidence after bariatric surgery is low. However, further studies with a larger sample size and longer follow-up duration are recommended to confirm our findings.

## Data Availability Statement

The raw data supporting the conclusion of this article will be available by the authors without undue reservation.

## Ethics Statement

This study received IRB approval (RC18/273/R) from King Abdullah International Medical Research Center (KAIMRC) at the Ministry of National Guard Health Affairs.

## Author Contributions

MA and GA contributed to the design and implementation of the research and the writing of the manuscript, with support from ES. OA analyzed the data and wrote the methodology. ES, NA, DA, HA, and AY collected the data and co-wrote the manuscript. AA, OA, and SE-B reviewed and edited the final manuscript. AA supervised the project. All authors agreed on the final manuscript.

## Conflict of Interest

The authors declare that the research was conducted in the absence of any commercial or financial relationships that could be construed as a potential conflict of interest.
